# Adverse Prognostic Impact of Postoperative Complications After Gastrectomy for Patients With Stage II/III Gastric Cancer: Analysis of Prospectively Collected Real-World Data

**DOI:** 10.3389/fonc.2021.611510

**Published:** 2021-04-29

**Authors:** Jeong Ho Song, Sejin Lee, Seohee Choi, Minah Cho, In Gyu Kwon, Yoo Min Kim, Taeil Son, Hyoung-Il Kim, Minkyu Jung, Woo Jin Hyung

**Affiliations:** ^1^ Department of Surgery, Yonsei University College of Medicine, Seoul, South Korea; ^2^ Gastric Cancer Center, Yonsei Cancer Center, Yonsei University Health System, Seoul, South Korea; ^3^ Department of Internal Medicine, Yonsei University College of Medicine, Seoul, South Korea

**Keywords:** gastric cancer, complication, chemotherapy, prognosis, real-world data (RWD)

## Abstract

**Background:**

The impact of postoperative complications on the prognosis of gastric cancer remains controversial. This study aimed to evaluate the relationship between postoperative complications and long-term survival in patients undergoing gastrectomy for stage II/III gastric cancer.

**Methods:**

Some 939 patients underwent curative gastrectomy for stage II/III gastric cancer were identified from real-world data prospectively collected between 2013 and 2015. We divided patients according to the presence of serious complications, specifically, Clavien-Dindo grade III or higher complications or those causing a hospital stay of 15 days or longer.

**Results:**

Serious complications occurred in 125 (13.3%) patients. Patients without serious complications (64.3%) completed adjuvant chemotherapy significantly more than patients with serious complications (37.6%; p<0.001). The 5-year overall survival(OS) rate was 58.1% and recurrence-free survival(RFS) rate was 58.1% in patients with serious complications, which were significantly worse than those of patients without serious complications (73.4% and 74.7%, respectively; p<0.001 for both). In stage II, once patients completed adjuvant chemotherapy adequately, the OS and RFS of patients with serious complications did not differ from those without serious complications. However, in stage III, the patients with serious complications showed a worse OS even after completion of adequate adjuvant chemotherapy.

**Conclusion:**

Serious complications after gastrectomy had a negative impact on the prognosis of stage II/III gastric cancer patients. Serious complications worsen the survival in association with inadequate adjuvant chemotherapy. Efforts to reduce serious complications, as well as support adequate chemotherapy through proper management of serious complications, would improve the prognosis of stage II/III gastric cancer patients.

## Introduction

Over a million new gastric cancer are diagnosed worldwide, making it the fifth most common cancer and the third leading cause of cancer death (1). Radical gastrectomy provides the only definitive chance to cure gastric cancer (2, 3); however, patients that undergo gastrectomy frequently experience postoperative complications. The complication rates following gastrectomy for gastric cancer is reported to be approximately 10–60% (4, 5), with 1.3–12.5% of major complications (6, 7), despite recent advances in surgical techniques and perioperative patient care.

Complications after cancer surgery have detrimental effects on the prognosis of cancer patients, which has been shown in colorectal cancer (8), head and neck cancer (9), and esophageal cancer (10). Several studies have evaluated the impact of postoperative complications on the long-term outcome of gastric cancer patients (5, 6, 11–13). However, the prognostic impact of complications after gastrectomy on gastric cancer patients remains controversial. These conflicting results are derived from the potential under-reporting of complications and their management in retrospective studies, as well as the selection bias of enrolling relatively physically fit patients in prospective studies. Data from a real-world setting may overcome these drawbacks and clarify the prognostic impact of complications after gastrectomy.

We hypothesized that the effect of complications after gastrectomy on the prognosis of gastric cancer patients could be exactly evaluated with prospectively collected real-world data. This study aimed to investigate the relationship between postoperative complications and long‐term survival in a large cohort of patients undergoing gastrectomy for gastric cancer using prospectively collected real-world data.

## Materials and Methods

### Patients

A prospectively collected gastric cancer database consisting of 3363 patients who underwent gastrectomy from January 2013 to December 2015 was reviewed retrospectively. A total of 1009 patients with pathologic stage II/III gastric cancer who underwent curative gastrectomy was identified from the database. Patients who met any of the following criteria were excluded from the analysis: completion total gastrectomy, gastrectomy combined with other cancer surgery, history of preoperative chemotherapy or radiotherapy, mortality or recurrence within 3 months of the operation, and lost to follow-up. No patient died of complications three months after gastrectomy. This study was approved by the Institutional Review Board of Severance Hospital, Yonsei University Health System (Protocol 4-2020-0303).

### Surgery

Seven surgeons performed gastrectomy for gastric cancer. Three of them performed only open surgery, whereas the other four surgeons performed minimally invasive surgery (MIS), including laparoscopic and robotic surgery, as well as open surgery. MIS was usually performed for patients with serosa-negative advanced gastric cancer with or without limited involvement of perigastric lymph nodes. In contrast, patients with serosa-positive advanced gastric cancer or extensive involvement of perigastric lymph nodes were generally considered for open gastrectomy. The gastric resection extent (total, distal subtotal, or proximal gastrectomy) was determined based on tumor location. D1+ lymphadenectomy was performed for early-stage gastric cancer and D2 lymphadenectomy was performed for advanced gastric cancer. The reconstruction method used for distal subtotal gastrectomy was gastroduodenostomy, gastrojejunostomy, or Roux-en Y gastrojejunostomy. The reconstruction method used for total gastrectomy was Roux-en Y esophagojejunostomy and that of proximal gastrectomy was double tract reconstruction (14). Tumor stage was defined according to the 8^th^ edition of American Joint Committee on Cancer staging system (15).

### Perioperative Management

At our institution, we established a “standard clinical pathway” for patients undergoing gastrectomy. We injected prophylactic antibiotics 15 minutes before surgery without administering routine postoperative antibiotics. We only used postoperative antibiotics in cases where gross bowel content contamination occurred during surgery or when symptoms and signs of inflammation, such as fever and leukocytosis, persisted for 3 days or more after surgery. We indwelled a urinary catheter in the operating room after anesthesia and removed it on postoperative day (POD) 1. Nasogastric tube was not inserted routinely, but rather only for patients with pyloric obstruction. Postoperative pain was primarily managed by patient-controlled anesthesia, and non-steroidal anti-inflammatory drugs were used for additional pain control. Intravenous antiemetics were injected every 12 hours only on the day of operation. Mucolytics were administered every 8 hours until POD 2 to support pulmonary toileting. Patients started drinking water on POD 2, having liquid diet on POD 3, and eating a soft diet on POD 4. On POD 5, patients were recommended to be discharged if ready.

Details of data collection and classification of postoperative complications are described in the **Supplementary Methods**.

### Adjuvant Chemotherapy

After surgery, patients with pathologic stage II/III gastric cancer were recommended to receive 5-FU-based adjuvant chemotherapy within 4–8 weeks after surgery. Old age itself was not a contraindication to adjuvant chemotherapy, but chemotherapy was not performed when the patient refused it or the patient’s performance status was poor. A majority of adjuvant chemotherapy regimens were TS-1 monotherapy or XELOX (capecitabine plus oxaliplatin), which were used to treat patients with stage II or stage III disease, respectively. TS-1 (80–120 mg per day) was administered for 4 weeks, followed by 2 weeks of rest. This 6-week regimen was repeated for eight cycles (16). The XELOX regimen involved 3-week cycles of oral capecitabine (1,000 mg/m^2^ twice daily on days 1–14 of each cycle) plus intravenous oxaliplatin (130 mg/m^2^ on day 1 of each cycle). The XELOX treatment regimen was administered for eight cycles (17).

### Follow Up

We followed up with all patients every 3 months for 1 year after surgery, and then every 6 months thereafter. They underwent abdomino-pelvic computed tomography (CT) scans every 6 months for 5 years after surgery. We performed an upper endoscopy every year. Recurrence was confirmed either by radiologic studies, such as CT, positron emission tomography, whole-body bone scan, or endoscopic examination with biopsy, or by surgery.

### Statistical Analysis

The serious complication (SC) group consisted of patients with Clavien-Dindo grade III or higher complications or patients with any complications causing a hospital stay of 15 days or longer. Patients without complications or who stayed in the hospital for less than 15 days with Clavien-Dindo grade I/II complications were defined as the non-serious complication (non-SC) group. We defined the adequate adjuvant chemotherapy group as patients who completed chemotherapy without omission or delayed initiation, while the inadequate adjuvant chemotherapy group as patients who omitted, delayed in initiation, or not completed the scheduled chemotherapy. Delayed initiation in adjuvant chemotherapy was defined as starting at 8 weeks after surgery (18), and incompletion of adjuvant chemotherapy was defined as discontinued treatment during the scheduled chemotherapy cycles.

Chi-square or Fisher’s exact tests for categorical variables and the Mann-Whitney test for continuous variables were used. The Kaplan-Meier method was used to calculate the overall and recurrence-free survival, whereas differences between survival curves were assessed using the log-rank test. The hazard ratio (HR) and two-sided 95% confidence intervals (CI) were estimated using a Cox proportional hazards model. A P-value less than 0.05 was considered statistically significant. All statistical analyses were performed using SPSS software for Windows (version 25.0; IBM Corp., Armonk, New York, USA).

## Results

### Patient Characteristics

A retrospective review of prospective data revealed 1009 patients who underwent curative gastrectomy for stage II/III gastric cancer. After 70 patients were excluded from the analyses (completion total gastrectomy [n=28]; combined other cancer surgery [n=17]; history for preoperative chemotherapy or radiotherapy [n=4]; mortality or recurrence within postoperative 3 months [n=17]; and follow-up loss [n=4]), data from 939 patients were included in this study.

Patient clinicopathologic characteristics are summarized in **Table 1**. The median age was 60 years (interquartile range (IQR), 52–70) and most patients were male (n=591; 62.9%). The median BMI was 22.9 (IQR 20.8–25.0) with 229 patients (24.4%) exhibiting a BMI of 25 or higher. The 68.5% of all patients (n=643) underwent open gastrectomy. The majority of patients underwent subtotal gastrectomy (n=641; 68.3%). This study consisted of 463 (49.3%) and 476 (50.7%) patients with pathologic stage II and stage III disease, respectively. Complications occurred in 741 out of 939 patients (78.9%), of which the number of patients with Clavien-Dindo grade I/II or grade III and higher was 655 (69.8%) or 86 (9.2%), respectively. (Additional details related to postoperative complications are in **Supplementary Table S2**). The non-SC group consisted of 198 patients without complications and 616 patients with complications less than 15 days of hospital stay. The SC group consisted of 86 patients with Clavien-Dindo grade III or higher and 39 patients with any complications causing a hospital stay of 15 days or longer. The SC group was more likely to be characterized by old age, male, and higher American Society of Anesthesiology (ASA) scores than the non-SC group (P=0.001, P=0.04, and P=0.021, respectively). The occurrence of serious complications was associated with tumors located in the upper body of the stomach or advanced tumor, which required total gastrectomy and open surgery rather than MIS (**Table 1**).

**Table 1:** Comparison of clinicopathologic characteristics according to serious complications.

	Overall(n=939)	Non-SC group(n=814)	SC group(n=125)	P-value
Age, median (IQR), years	60 (52–70)	60 (52–60)	66 (54–74)	0.001
Sex				0.04
Female	348 (37.1%)	312 (38.3%)	36 (28.8%)	
Male	591 (62.9%)	502 (61.7%)	89 (71.2%)	
BMI, median (IQR), kg/m^2^	22.9 (20.8–25.0)	22.8 (20.8–25.0)	23.3 (20.6–25.4)	0.477
ASA score				0.021
I	153 (16.3%)	137 (16.8%)	16 (12.8%)	
II	569 (60.6%)	502 (61.7%)	67 (53.6%)	
III	209 (22.3%)	169 (20.8%)	40 (32.0%)	
IV	8 (0.9%)	6 (0.7%)	2 (1.6%)	
Operation method				<0.001
Open	643 (68.5%)	534 (65.6%)	109 (87.2%)	
Laparoscopy	189 (20.1%)	180 (22.1%)	9 (7.2%)	
Robot	107 (11.4%)	100 (12.3%)	7 (5.6%)	
Surgical procedure				0.001
STG	641 (68.3%)	573 (70.4%)	68 (54.4%)	
TG	294 (31.3%)	238 (29.2%)	56 (44.8%)	
PG	4 (0.4%)	3 (0.4%)	1 (0.8%)	
Lymph node dissection				0.129
<D2	105 (11.2%)	96 (11.8%)	9 (7.2%)	
D2	834 (88.8%)	718 (88.2%)	116 (92.8%)	
Combined operation				<0.001
No	788 (83.9%)	703 (86.4%)	85 (68.0%)	
Yes	151 (16.1%)	111 (13.6%)	40 (32.0%)	
Histology				0.085
Differentiated	305 (32.5%)	256 (31.4%)	49 (39.2%)	
Undifferentiated	634 (67.5%)	558 (68.8%)	76 (60.8%)	
Tumor depth				0.043
T1	56 (6.0%)	52 (6.4%)	4 (3.2%)	
T2	130 (13.8%)	116 (14.3%)	14 (11.2%)	
T3	341 (36.3%)	294 (36.1%)	47 (37.6%)	
T4a	399 (42.5%)	344 (42.3%)	55 (44.0%)	
T4b	13 (1.4%)	8 (1.0%)	5 (4.0%)	
Lymph node metastasis				0.682
N0	226 (24.1%)	201 (24.7%)	25 (20.0%)	
N1	187 (19.9%)	159 (19.5%)	28 (22.4%)	
N2	241 (25.7%)	208 (25.6%)	33 (26.4%)	
N3	285 (30.4%)	246 (30.2%)	39 (31.2%)	
Pathologic stage^a^				0.097
II	463 (49.3%)	410 (50.4%)	53 (49.6%)	
III	476 (50.7%)	404 (42.4%)	72 (57.6%)	
Complication				
No	198 (21.1%)	198 (24.3%)	0	
Yes	741 (78.9%)	616 (75.7%)	125 (100%)	
Clavien-Dindo grade				
I	363 (49.0%)	359 (58.3%)	4 (3.2%)	
II	292 (39.4%)	257 (41.7%)	35 (28.0%)	
III	76 (10.3%)	0	76 (60.8%)	
IV	10 (1.3%)	0	10 (8.0%)	

SC, serious complications; IQR, interquartile range; BMI, body mass index (calculated as weight in kilograms divided by height in meters squared); ASA, American Society of Anesthesiology; STG, subtotal gastrectomy; TG, total gastrectomy; PG, proximal gastrectomy.

a Pathologic stages were defined in accordance with the 8th edition of American Joint Committee on Cancer staging system.

Compared with the non-SC group, patients who stayed in the hospital for 15 days or longer with Clavien-Dindo grade I/II complications were significantly associated with old age, high ASA score, open surgery, and combined operation (P=0.040, P=0.025, P<0.001, and P=0.011, respectively; **Supplementary Table S3**). The clinicopathologic characteristics of patients with Clavien-Dindo grade I/II complications causing a hospital stay of 15 days or longer were similar to those with Clavien-Dindo grade III or higher complications, except for histology (**Supplementary Table S4**). The median hospital stays of patients with Clavien-Dindo grade I/II complications causing a hospital stay of 15 days was 16 days (IQR 15–22), which was similar to that of patients with Clavien-Dindo grade III or higher complications (median 15; IQR 10–22). The median stay for the non-SC group was 7 days (IQR 6–8).

### Relationship Between Postoperative Complication and Adjuvant Chemotherapy

Overall, 750 of 939 (79.9%) patients were treated with adjuvant chemotherapy. The SC group was associated with a higher rate of omission (n=41, 32.8%) than the non-SC group (n=148, 18.2%; P<0.001). The SC group also exhibited more instances of delayed initiation, incompletion, and inadequacy of adjuvant chemotherapy (n=8, 9.5%; n=32, 38.1%; n=78, 62.4%) than the non-SC group (n=14, 2.1%, P=0.002; n=135, 20.3%, P<0.001; n=291, 35.7%, P<0.001, respectively) (**Table 2**). Similar findings were observed in stages II and III (**Supplementary Table S5**, **S6**), with only marginal differences seen in the number of patients who did not complete their chemotherapy.

**Table 2:** Relationship between postoperative complications and adjuvant chemotherapy.

Adjuvant chemotherapy	Overall (n=939)	Non-SC group (n=814)	SC group (n=125)	P-value
Omission	939			<0.001
(-)	750 (79.9%)	666 (81.8%)	84 (67.2%)	
(+)	189 (20.1%)	148 (18.2%)	41 (32.8%)	
Time to AC initiation	750			0.002
≤8 weeks	728 (97.1%)	652 (97.9%)	76 (90.5%)	
>8 weeks (Delay)	22 (2.9%)	14 (2.1%)	8 (9.5%)	
Scheduled cycles of AC	750			<0.001
Completed	583 (77.7%)	531 (79.7%)	52 (61.9%)	
Not completed	167 (22.3%)	135 (20.3%)	32 (38.1%)	
Adequacy of AC	939			<0.001
Adequate^a^	570 (60.7%)	523 (64.3%)	47 (37.6%)	
Inadequate^b^	369 (39.3%)	291 (35.7%)	78 (62.4%)	

SC, serious complications; AC; adjuvant chemotherapy.

a Adequate AC was defined when AC was completed without omission or delayed initiation.

b Inadequate AC was defined when AC was omitted, delayed initiation, or not completed the scheduled chemotherapy cycles.

The rate of omission and inadequacy of adjuvant chemotherapy were significantly higher in Clavien-Dindo grade I/II patients who stayed 15 days or longer in the SC group compared to the non-SC group (**Supplementary Table S7**). However, the rate of omission, delayed initiation, incompletion and inadequacy of adjuvant chemotherapy were similar between the Clavien-Dindo grade I/II patients who stayed 15 days or longer and those with Clavien-Dindo grade III or higher complications (**Supplementary Table S8**).

### Survival Outcomes

**Figure 1:**
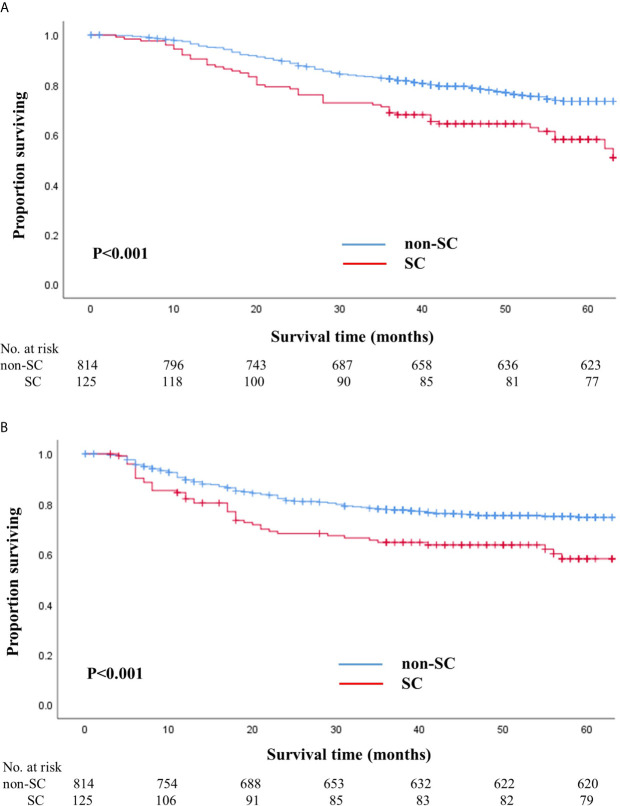
Overall **(A)** and recurrence-free **(B)** survival in patients with and without serious complications. **(A)** Overall survival in patients with and without serious complications. **(B)** Recurrence-free survival in patients with and without serious complications.

The median follow-up duration after surgery was 52 months (range: 7–71 months) until the cutoff date of December 31, 2018. During the follow-up period, 245 patients (26.1%) died, of which 52 (41.6%) were from the SC group and 193 (23.7%) were from the non-SC group. The HR for death in the SC group, as compared with that in the non-SC group, was 1.92 (95% CI, 1.4–2.6; P<0.001). The 5-year overall survival rate of the SC group was 58.1% (95% CI, 49.2–68.6) and that of the non-SC group was 71.3% (95% CI, 70.0–76.9; log-rank P<0.001) (**Figure 1**).

The number of patients that experienced a recurrence was 240 (25.6%), of which 58 (46.4%) were from the SC group and 182 (22.4%) were from the non-SC group. The HR for recurrence in the SC group, as compared to the non-SC group, was 1.75 (95% CI, 1.3–2.4; P=0.001). The 5-year recurrence-free survival rate in SC patients was 58.1% (95% CI, 49.0–69.0) and that in non-SC patients was 74.7% (95% CI, 71.5–77.9; log-rank P<0.001) (**Figure 1**). When we stratified the patients according to pathologic stages, the SC group exhibited worse overall and recurrence-free survival rates than the non-SC group for patients with stages II and III (**Supplementary Figure S1**).

**Figure 2:**
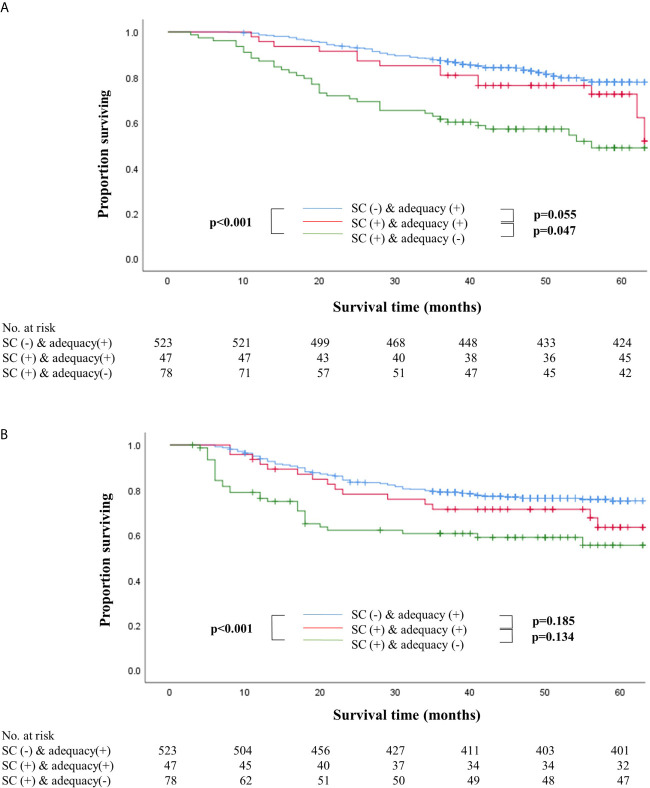
Overall **(A)** and recurrence-free **(B)** survival according to the presence of serious complications with adequacy of adjuvant chemotherapy. **(A)** Overall survival according to the presence of serious complications with adequacy of adjuvant chemotherapy. **(B)** Recurrence-free survival according to the presence of serious complications with adequacy of adjuvant chemotherapy.

Survival outcomes were also compared after dividing the patients into three groups, namely non-SC patients who received adequate chemotherapy (n=523), SC patients who received adequate chemotherapy (n=47), and SC patients whose chemotherapy was inadequate (n=78). Patients who received inadequate chemotherapy in the SC group showed significantly worse overall and recurrence-free survival than non-SC patients who received adequate chemotherapy (log-rank P<0.001 for both) (**Figure 2**). In stage II, SC patients who received adequate chemotherapy had similar overall and recurrence-free survival outcomes to those under adequate chemotherapy in the non-SC group (log-rank P=0.495 and P=0.936, respectively). On the contrary, in stage III, the overall and recurrence-free survival of SC patients under adequate chemotherapy were similar to those who did not receive adequate chemotherapy in the SC group (log-rank P=0.426 and P=0.551, respectively). Non-SC patients under adequate chemotherapy had significantly higher overall survival than SC patients who received adequate chemotherapy (log-rank P=0.013) even though the recurrence-free survival did not differ statistically (log-rank P=0.115) (**Supplementary Figure S2**).

Patients with Clavien-Dindo grade I/II complications causing a hospital stay of 15 days or longer exhibited worse overall and recurrence-free survival than the non-SC group (**Supplementary Figure S3**). In the SC group, the overall and recurrence-free survival of patients with Clavien-Dindo grade I/II complications causing a hospital stay of 15 days or longer were similar to those of patients with Clavien-Dindo grade III or higher complications (**Supplementary Figure S4**).

Multivariable analyses of overall and recurrence-free survival identified that serious complication was not an independent risk factor among patients under adequate adjuvant chemotherapy (HR, 1.53; 95% CI, 0.9–2.6, P=0.129 for overall survival; HR, 1.32; 95% CI, 0.8–2.3, P=0.310 for recurrence-free survival; **Table 3**). Stratified by stages, inadequate adjuvant chemotherapy was not a risk factor among patients without serious complications (HR, 1.45; 95% CI, 0.8-2.6, P=0.215; **Table 4**) in the multivariable analysis of recurrence-free survival in stage II. The presence of serious complications was a risk factor for overall survival among patients under adjuvant chemotherapy in stage III (HR, 1.82; 95% CI, 1.0–3.3, P=0.043; **Table 5**).

**Table 3:** Multivariate analyses of overall and recurrence-free survival in stage II-III gastric cancer patients.

Characteristics	No. of patients	Overall survival			Recurrence-free survival		
		HR	95% CI	*P*-value	HR	95% CI	*P*-value
Age				0.029			0.536
≤60 years	471	1			1		
>60 years	468	1.347	1.030–1.762		1.089	0.830–1.429	
Sex				0.773			0.691
Female	348	1			1		
Male	591	1.040	0.797–1.358		0.948	0.728–1.234	
ASA score				0.906			0.066
I or II	722	1			1		
III or IV	217	1.018	0.759–1.366		0.734	0.528–1.021	
Operation method				0.097			0.443
MIS	296	1			1		
Open	643	1.316	0.952–1.819		1.138	0.818–1.582	
Resection extent							
Distal subtotal	641	1			1		
Total	294	1.066	0.716–1.588	0.753	1.300	1.000–1.689	0.050
Proximal	4	1.322	0.165–10.583	0.793	N/A	N/A	0.946
Lymph node dissection				0.642			0.087
Less than D2	105	1			1		
D2	834	1.126	0.683–1.854		1.713	0.925–3.169	
Tumor size				<0.001			<0.001
≤50 mm	543	1			1		
>50 mm	396	1.682	1.292–2.189		1.849	1.414–2.417	
Tumor location							
Lower	483	1			1		
Middle	248	1.268	0.924–1.739	0.142	0.996	0.711–1.394	0.980
Upper/Whole	208	1.257	0.930–1.701	0.137	1.152	0.741–1.792	0.529
Histology				0.925			0.821
Differentiated	305	1			1		
Undifferentiated	634	1.014	0.757–1.359		0.966	0.713–1.307	
LVI				0.547			0.426
(-)	369	1			1		
(+)	570	1.093	0.818–1.460		1.129	0.838–1.521	
SC & AC							
SC (-) & adequate^a^ AC	523	1			1		
SC (+) & adequate AC	47	1.526	0.884–2.635	0.129	1.323	0.772–2.266	0.308
SC (-) & inadequate^b^ AC	291	2.293	1.709–3.078	<0.001	1.599	1.190–2.149	0.002
SC (+) & inadequate AC	78	2.669	1.807–3.942	<0.001	2.273	1.524–3.388	<0.001
Pathologic TNM stage				<0.001			<0.001
II	463	1			1		
III	476	3.197	2.370–4.312		3.137	2.306–4.268	

HR, hazard ratio; CI, confidence interval; ASA, American Society of Anesthesiology; MIS, minimally invasive surgery; N/A, not applicable; LVI, lymphovascular invasion; SC, Serious complications; AC adjuvant chemotherapy.

a Adequate AC was defined when AC was completed without omission or delayed initiation.

b Inadequate AC was defined when AC was omitted, delayed in initiation, or not completed the scheduled chemotherapy cycles.

**Table 4:** Multivariate analyses of overall and recurrence-free survival in stage II gastric cancer patients.

Characteristics	No. of patients	Overall survival			Recurrence-free survival		
		HR	95% CI	*P*-value	HR	95% CI	*P*-value
Age				0.468			0.142
≤60 years	239	1			1		
>60 years	224	1.222	0.711–2.102		0.656	0.373–1.152	
Sex				0.508			0.651
Female	164	1			1		
Male	299	1.212	0.686–2.140		0.878	0.499–1.545	
ASA score				0.955			0.044
I or II	355	1			1		
III or IV	108	1.017	0.565–1.830		0.468	0.224–0.981	
Operation method				0.909			0.603
MIS	190	1			1		
Open	273	0.967	0.543–1.722		1.168	0.650–2.099	
Resection extent							
Distal subtotal	343	1			1		
Total	117	1.733	1.034–2.904	0.037	1.521	0.672–3.442	0.314
Proximal	3	0.974	N/A	0.974	N/A	N/A	0.973
Lymph node dissection				0.534			0.256
Less than D2	80	1			1		
D2	383	1.274	0.593–2.737		1.725	0.674–4.415	
Tumor size				0.033			0.001
≤50 mm	328	1			1		
>50 mm	135	1.724	1.043–2.848		2.346	1.393–3.954	
Tumor location							
Lower	236	1			1		
Middle	136	0.661	0.336–1.301	0.230	0.710	0.361–1.394	0.319
Upper/Whole	91	0.997	0.412–2.415	0.995	1.732	0.944–3.176	0.076
Histology				0.484			0.845
Differentiated	163	1			1		
Undifferentiated	300	1.223	0.697–2.146		1.062	0.581–1.944	
LVI				0.904			0.733
(-)	263	1			1		
(+)	200	0.967	0.562–1.665		1.108	0.616–1.991	
SC & AC							
SC (-) & adequate^a^ AC	241	1			1		
SC (+) & adequate AC	20	0.461	0.062–3.427	0.449	0.785	0.184–3.347	0.744
SC (-) & inadequate^b^ AC	169	1.913	1.089–3.361	0.024	1.445	0.808–2.584	0.215
SC (+) & inadequate AC	33	4.471	2.238–8.933	<0.001	4.317	2.005–9.294	<0.001

HR, hazard ratio; CI, confidence interval; ASA, American Society of Anesthesiology; MIS, minimally invasive surgery; N/A, not applicable; LVI, lymphovascular invasion; SC, Serious complications; AC adjuvant chemotherapy.

a Adequate AC was defined when AC was completed without omission or delayed initiation.

b Inadequate AC was defined when AC was omitted, delayed in initiation, or not completed the scheduled chemotherapy cycles.

**Table 5:** Multivariate analyses of overall and recurrence-free survival in stage III gastric cancer patients.

Characteristics	No. of patients	Overall survival			Recurrence-free survival		
		HR	95% CI	*P*-value	HR	95% CI	*P*-value
Age				0.038			0.181
≤60 years	232	1			1		
>60 years	244	1.390	1.018–1.898		1.237	0.906–1.688	
Sex				0.739			0.588
Female	184	1			1		
Male	292	0.948	0.692–1.299		0.919	0.676–1.248	
ASA score				0.810			0.273
I or II	367	1			1		
III or IV	109	0.958	0.678–1.356		0.813	0.561–1.178	
Operation method				0.083			0.541
MIS	106	1			1		
Open	370	1.442	0.953–2.180		1.133	0.759–1.689	
Resection extent							
Distal subtotal	298	1			1		
Total	177	0.882	0.555–1.402	0.596	1.200	0.889–1.618	0.233
Proximal	1	2.577	0.311–21.371	0.381	N/A	N/A	0.949
Lymph node dissection				0.898			0.146
Less than D2	25	1			1		
D2	451	1.044	0.541–2.014		1.830	0.810–4.134	
Tumor size				0.001			<0.001
≤50 mm	215	1			1		
>50 mm	261	1.705	1.252–2.322		1.876	1.381–2.549	
Tumor location							
Lower	247	1			1		
Middle	112	1.516	1.055–2.179	0.025	1.153	0.784–1.695	0.469
Upper/Whole	117	1.164	0.819–1.655	0.397	1.117	0.674–1.851	0.667
Histology				0.432			0.621
Differentiated	142	1			1		
Undifferentiated	334	0.876	0.628–1.220		0.916	0.648–1.295	
LVI				0.610			0.391
(-)	106	1			1		
(+)	370	1.100	0.763–1.586		1.175	0.813–1.700	
SC & AC							
SC (-) & adequate^a^ AC	282	1			1		
SC (+) & adequate AC	27	1.821	1.020–3.250	0.043	1.465	0.821–2.615	0.196
SC (-) & inadequate^b^ AC	122	2.561	1.825–3.594	<0.001	1.622	1.153–2.282	0.005
SC (+) & inadequate AC	45	2.246	1.401–3.599	0.001	1.791	1.117–2.871	0.016

HR, hazard ratio; CI, confidence interval; ASA, American Society of Anesthesiology; MIS, minimally invasive surgery; N/A, not applicable; LVI, lymphovascular invasion; SC, Serious complications; AC adjuvant chemotherapy.

a Adequate AC was defined when AC was completed without omission or delayed initiation.

b Inadequate AC was defined when AC was omitted, delayed in initiation, or not completed the scheduled chemotherapy cycles.

Of the 240 patients with recurrence, there was no difference in recurrence pattern between the two groups (**Table 6**). The peritoneum was the most frequent initial site of recurrence in both groups. SC group had more frequent hematogenous metastasis (n=11, 23.9%) than the non-SC group (n=31, 16.0%), although it was not a statistically significant difference (P=0.203).

**Table 6:** Comparison of initial recurrence site according to serious complications in patients with recurrence.

Recurrence pattern	Overall(n=240)	Non-SC group(n=194)	SC group(n=46)	P-value
Locoregional	23 (9.6%)	21 (10.8%)	2 (4.3%)	0.266
Peritoneal	104 (43.3%)	85 (43.8%)	19 (41.3%)	0.757
Hematogenous	42 (17.5%)	31 (16.0%)	11 (23.9%)	0.203
Distant lymph node	32 (13.3%)	27 (13.9%)	5 (10.9%)	0.585
mixed	39 (16.3%)	30 (15.5%)	9 (19.6%)	0.498

SC, serious complications.

## Discussion

In this study, patients with serious complications demonstrated worse survival and a higher rate of disease recurrence than patients without serious complications after curative gastrectomy for stage II/III gastric cancer. A majority of patients with serious complications failed to receive adequate adjuvant therapy. Eventually, patients who experienced serious complications combined with inadequate adjuvant chemotherapy revealed the worst survival rate as well as the highest recurrence rates.

Randomized controlled studies have established that adjuvant chemotherapy following gastrectomy has survival advantages compared to gastrectomy alone (16, 17). Thus, adequate delivery and completion of chemotherapy is necessary to obtain a survival benefit after curative gastrectomy for stage II/III gastric cancer. Poor survival of patients with postoperative complications after gastrectomy for stage II/III gastric cancer cannot be considered apart from adjuvant chemotherapy. As shown in this study, the SC group demonstrated a higher proportion of patients who did not receive adequate chemotherapy.

Consistent with our results, postoperative complications in gastric cancer patients prompted failure to complete multimodal therapy even in a perioperative chemotherapy setting (19). Patients are relatively tolerant of perioperative chemotherapy because they receive chemotherapy under a relatively healthy condition compared to a postoperative condition. Therefore, it is difficult to determine the effect of postoperative complications on the survival outcomes of patients who have received preoperative chemotherapy because chemotherapy itself can affect postoperative complications and survival outcomes. Moreover, in the perioperative chemotherapy setting, all patients completed preoperative chemotherapy even though postoperative chemotherapy could be affected by postoperative complications. However, in the adjuvant setting, patients are affected by the effect of the complication on the whole process of chemotherapy. Thus, adverse effect of complications on chemotherapy would be greater in an adjuvant setting than in a perioperative one.

Studies analyzed the relationship among postoperative complications, chemotherapy, and survival outcomes demonstrated that survival outcomes of patients with complications who received adjuvant therapy or completed multimodal therapy were comparable to those of patients without complications, similar to this study. The prognostic impact of serious complications differed according to pathologic stage in our study. Assessing the impact of serious complications combined with the adequacy of chemotherapy on survival outcome is very complex. In this study, the presence of serious complications in stage III patients resulted in poor prognosis regardless of adequacy of chemotherapy. Since patients tolerate better with preoperative chemotherapy than postoperative adjuvant chemotherapy, preoperative chemotherapy has a higher completion rate than postoperative adjuvant chemotherapy. Preoperative chemotherapy does not increase the incidence of surgical complications (19, 20). Furthermore, the survival outcomes after preoperative chemotherapy were similar between patients who experienced postoperative complications and those who did not (21). Thus, preoperative chemotherapy may be considered for patients who are more likely to develop complications, especially when stage III is suspected by preoperative assessments. However, the adverse impact of serious complications was not profound in stage II patients. If chemotherapy was delivered adequately, even patients with serious complications in stage II demonstrated similar survival to those who did not experience serious complications. Thus, it is important to treat serious complications befittingly to administer adequate chemotherapy.

To consider the timely treatment of complications, we combined the length of stay and the severity of Clavien-Dindo complication grade to define serious complications. Most previous studies in gastric cancer used a modified Clavien-Dindo classification system, and grade III or higher was used to express the severity of the complication (6, 22, 23). Under this classification, many surgeons expect patients with complications to recover from conservative care without active treatment to avoid a high-grade complication rating. As a result, patients stay longer in the hospital and have poor general conditions, which is most likely adversely affects the delivery of chemotherapy and survival outcomes even without serious complications. Furthermore, patients remaining in the hospital for 15 days or longer with Clavien-Dindo grade I/II complications showed similar clinicopathologic characteristics as those with Clavien-Dindo grade III or higher complications. Similar impact on the adequacy of chemotherapy and survival outcomes was also observed. Therefore, serious complications are more suitable in determining patient group according to the severity of postoperative complications.

The impacts of postoperative complications on the long-term survival outcomes of cancer patients are well documented (8–10). Complication worsens the prognosis of patients with gastrointestinal cancer, including gastric cancer. The adverse effects of serious complications on prognosis in our study are consistent with the previous studies (5, 11, 19, 20). The poor prognosis of patients with serious complications was presumed since host immunosuppression and proinflammatory cytokines induce the growth of residual cancer cells (24, 25). In fact, patients who experienced anastomotic leakage, intra-abdominal infectious complications, or inflammatory complications following gastrectomy demonstrated worse survival than those without complications (5, 11, 26).

This study has several limitations. First, only a relatively small number of patients with serious complications limited to analyze the impact of the type of complications on adjuvant chemotherapy and survival outcomes. It is necessary to identify the effect of each type of complication on prognosis after gastrectomy. Second, the regimens of chemotherapy used in this study were not homogeneous, although most of patients received either TS-1 monotherapy or XELOX. Differences in chemotherapy regimen may influence the adequacy because they affect patient compliance. It is difficult to assess the precise relationship between postoperative complications and adequacy of chemotherapy with patient prognosis. Another possible limitation is the retrospective nature of this study. To exactly evaluate the prognostic impact of complications together with chemotherapy, a randomized trial would be ideal. However, a randomized clinical trial is not always feasible in complication-related research. Moreover, the patient population selected by the inclusion and exclusion criteria in a prospective study is relatively not vulnerable, resulting in fewer postoperative complications which do not reflect real clinical practice. Another pitfall of randomized clinical trials is the possibility of a selection bias in which only patients who are fit and give informed consent to participate in the study are enrolled. Therefore, patients enrolled in the randomized clinical trials of surgical procedures are relatively in good general condition. As a result, the real-world study is considered to have more generalizability compared to randomized clinical trials. Therefore, in this study, we used prospectively collected real-world data which reflected real clinical practice.

Our study confirmed that serious complications after gastrectomy had a negative impact on the prognosis of patients with stage II/III gastric cancer through a prospectively collected data analysis. Serious complications significantly worsened survival outcomes when inducing inadequacy of adjuvant chemotherapy, rather than complication themselves. Therefore, efforts should be made to reduce serious complications as well as support adequate chemotherapy in a timely manner through the proper management of serious complications, which will improve the long-term survival of patients with stage II/III gastric cancer. In addition, perioperative chemotherapy could be an alternative option for patients with a high risk of serious complications, even in Eastern countries where adjuvant chemotherapy is a standard treatment for patients with stage II/III gastric cancer.

## Data Availability Statement

The datasets presented in this article are not readily available because additional study is planned with the dataset used for this article. Requests to access the datasets should be directed to WH, wjhyung@yuhs.ac.

## Ethics Statement

The studies involving human participants were reviewed and approved by Institutional Review Board of Severance Hospital, Yonsei University Health System (Protocol 4-2020-0303). The patients/participants provided their written informed consent to participate in this study.

## Author Contributions

JS and WH designed the study. The acquisition, analysis or interpretation of the data was conducted by JS, SL, SC, and WH. MC, YK, TS, H-IK, and WH made contribution in administrative, technical, or material support. Critical revision of the manuscript for important intellectual content was conducted by IK and MJ. All authors contributed to the article and approved the submitted version.

## Funding

WH has stock in Hutom, received a research grant from Medtronic (4-2017-0940) and GC Pharma (4-2018-0306), and was a consultant for Ethicon and Verb Surgical. The funding sources had no role in the design and conduct of the study; collection of data, analysis and interpretation; preparation, review or approval of the manuscript; or decision to submit the manuscript for publication.

## Conflict of Interest

WH reports receiving research grants from the Medtronic and GC Pharma, is the chief executive officer of Hutom, and holds its stock. He provided consultancy services to Ethicon and Verb Surgical outside of the submitted work.

The remaining authors declare that the research was conducted in the absence of any commercial or financial relationships that could be construed as a potential conflict of interest.
