# Supplementary material for: Adverse Prognostic Impact of Postoperative Complications After Gastrectomy for Patients With Stage II/III Gastric Cancer: Analysis of Prospectively Collected Real-World Data
Source: Front Oncol. 2021 Apr 29;11:611510. doi: 10.3389/fonc.2021.611510 (PMC8116792; doi:10.3389/fonc.2021.611510)
Supplement: Supplementary file 1 [file DataSheet_1.docx]

**Supplementary material**

**Supplementary Methods**

**Table S1.** Definition of the complications.

**Table S2.** Type and severity of postoperative complications.

**Table S3.** Comparison of clinicopathologic characteristics between the non-SC group and patients with a hospital stay of 15 days or longer with a Clavien-Dindo grade I/II.

**Table S4.** Comparison of clinicopathologic characteristics between patients with a hospital stay of 15 days or longer with a Clavien-Dindo grade I/II and those with a Clavien-Dindo grade III or higher.

**Table S5.** Relationship between postoperative complications and adjuvant chemotherapy in stage II gastric cancer.

**Table S6.** Relationship between postoperative complications and adjuvant chemotherapy in stage III gastric cancer.

**Table S7.** Comparison of adequacy of adjuvant chemotherapy between the non-SC group and the patients with a hospital stay of 15 days or longer with Clavien-Dindo I/II in stage II/III gastric cancer.

**Table S8.** Relationship between Clavien-Dindo grade and adjuvant chemotherapy in stage II/III gastric cancer patients with serious complications.

**Figure S1.** Overall (A, C) and recurrence-free survival (B, D) in patients with and without serious complications after gastrectomy by pathologic stage. (A, B) Pathologic stage II. (C, D) Pathologic stage III.

**Figure S2.** Overall (A, C) and recurrence-free survival (B, D) according to the presence of serious complications with adequacy of adjuvant chemotherapy by pathologic stage. (A, B) Pathologic stage II. (C, D) Pathologic stage III.

**Figure S3.** Overall (A) and recurrence-free survival (B) in non-SC group and patients with a hospital stay of 15 days or longer with a Clavien-Dindo grade I/II.

**Figure S4.** Overall (A) and recurrence-free survival (B) in patients with Clavien-Dindo grade III or higher and those with a hospital stay of 15 days or longer with a Clavien-Dindo grade I/II.

**Supplementary Methods**

**Data collection and classification of postoperative complications**

Our institution has a clinical data retrieval program that allows researchers to search and organize patient electronic medical records without manual intervention by the researchers. As such, data accuracy is maximized and missing data are minimized. Using this program, we organized a prospective database of patient demographics, operative outcomes, pathologic features, and survival outcomes. We also extracted all medications, radiologic, laboratory, and body temperature data through this program to confirm whether any complications occurred.

To obtain well-organized patient complication data, six or more surgeons held weekly meetings to confirm postoperative complications, discuss treatment plans, and evaluate treatment efficacy for complications (Supplementary Table S1). The complications included re-admission due to any surgery-related complications within 90 days of surgery. We used the modified Clavien-Dindo classification to grade the severity of complications [1]. Most patients exhibiting a grade II complication in our study were those who were taking medications, such as liver- or pancreas-supporting drugs, antipyretics, or antibiotics to treat elevated hepatic or pancreatic enzymes or fever. Patients with grade I complications did not take any medications but exhibited abnormal chest radiograph findings, such as atelectasis and pleural effusion, or a body temperature above 38°C.

**Table 1.** Definition of the complications

| **Complication type** | **Definition** |
| --- | --- |
| Surgical complications | |
| Fluid collection | Loculated fluid collection confirmed by imaging modality |
| Intra-abdominal abscess | Collection of pus or infected fluid collection caused by bacteria |
| Chyle leakage | Diagnosed as the presence of milky fluid in a drain with a triglyceride level ≥110 mg/dl |
| Intra-abdominal bleeding | Bleeding confirmed by CT scan or abdominal drains |
| Intra-luminal bleeding | Bleeding confirmed by nasogastric tube drainage or endoscopy |
| Intestinal obstruction | Defined as mechanical obstruction with an air-fluid level on imaging studies |
| Ileus | Temporary impairment of gastrointestinal motility following surgery (72 hours) without any stenosis, obstruction, or inflammation |
| Gastric stasis | Delayed emptying of stomach in the absence of mechanical obstruction confirmed by imaging modality |
| Anastomotic stenosis | Inability to pass the anastomotic site with endoscope |
| Anastomotic leakage | Diagnosed when the luminal contents were detected in a drain or a leak was confirmed by imaging modality |
| Pancreatitis | Elevated amylase of percutaneous drain ( 3 times the upper normal limit of serum amylase) starting from postoperative day 3 or elevated serum amylase treated by octreotide or camostat mesilate |
| Remnant stomach ischemia | Insufficient blood flow to remnant stomach confirmed by contrast CT scans |
| Anemia | Decrease of hemoglobin in blood requiring transfusion or iron supply |
| Wound complication | Seroma, hematoma, infection, or dehiscence of the wound |
| Medical complications | |
| Pulmonary | Acute respiratory failure requiring intervention or atelectasis, pleural effusion, or pneumonia in a chest X-ray or CT scan read by radiologists |
| Urinary | Voiding difficulty or urinary tract infection |
| Hepatic | Elevated liver enzymes treated by hepatotonics or cholecystitis confirmed by imaging modality |
| Cardiac | Hypertension, arrhythmia, angina, or myocardial infarction |
| Fever | Defined as body temperature above 38°C. If body temperature was less than 38°C, patients were prescribed antipyretics and regarded as having complication grade I or were regarded as having complication grade II if antibiotics were prescribed. Patients with body temperature over 38°C who recovered without antibiotics or antipyretics were classified as having complication grade I. |

*CT, computed tomography.*

**Table 2.** Type and severity of postoperative complications

| **Type of complications** | **Overall (N=741)** | **CD grade I/II/III/IV** | **Minor^a^ complications (N=616)** | **Serious^b^ complications (N=125)** |
| --- | --- | --- | --- | --- |
| **Medical complications** | **473** | **326/125/18/4** | **446 (72.4%)** | **27 (21.6%)** |
| Pulmonary | 181 | 130/29/18/4 | 157 (25.4%) | 24 (19.2%) |
| Hepatic | 56 | 0/56/0/0 | 55 (8.9%) | 1 (0.8%) |
| Urinary | 20 | 3/17/0/0 | 19 (3.1%) | 1 (0.8%) |
| Cardiac | 5 | 0/5/0/0 | 4 (0.6%) | 1 (0.8%) |
| Fever | 202 | 191/11/0/0 | 202 (32.8%) | 0 |
| Others | 9 | 2/7/0/0 | 9 (1.5%) | 0 |
| **Surgical complications** | **268** | **37/167/58/6** | **170 (27.6%)** | **98 (78.4%)** |
| Wound problem | 36 | 23/11/2/0 | 30 (4.9%) | 6 (4.8%) |
| Intra-luminal bleeding | 2 | 0/2/0/0 | 2 (0.3%) | 0 |
| Intra-abdominal abscess | 5 | 0/1/2/2 | 0 | 5 (4.0%) |
| Fluid collection | 14 | 2/10/2/0 | 6 (1.0%) | 8 (6.4%) |
| Chyle leakage | 47 | 4/15/28/0 | 16 (2.6%) | 31 (24.8%) |
| Anastomotic leakage | 20 | 0/6/13/1 | 1 (0.2%) | 19 (15.2%) |
| Intra-abdominal bleeding | 8 | 0/1/4/3 | 1 (0.2%) | 7 (5.6%) |
| Ileus | 3 | 1/2/0/0 | 2 (0.3%) | 1 (0.8%) |
| Intestinal obstruction | 9 | 1/5/3/0 | 3 (0.5%) | 6 (4.8%) |
| Anastomotic stenosis | 6 | 0/2/4/0 | 2 (0.3%) | 4 (3.2%) |
| Pancreatic complication | 55 | 0/55/0/0 | 48 (7.8%) | 7 (5.6%) |
| Gastric stasis | 5 | 2/3/0/0 | 5 (0.8%) | 0 |
| Remnant stomach ischemia | 4 | 0/4/0/0 | 3 (0.5%) | 1 (0.8%) |
| Anemia | 54 | 4/50/0/0 | 51 (8.3%) | 3 (2.4%) |

*CD, Clavien-Dindo.*

*^a^Minor complications were defined as complications requiring a hospital stay less than 15 days with a Clavien-Dindo grade I/II.*

*^b^Serious complications were defined as Clavien-Dindo grade III or higher complications or complications causing a hospital stay of 15 days or longer.*

**Table 3.** Comparison of clinicopathologic characteristics between the non-SC group and patients with a hospital stay of 15 days or longer with a Clavien-Dindo grade I/II

|  | **Overall (n=853)** | **Non-SC group (n=814)** | **Hospital stay ≥15 days with CD I/II (n=39)** | **P-value** |
| --- | --- | --- | --- | --- |
| Age, median (IQR), years | 60 (52–69) | 60 (52–69) | 64 (56–71) | 0.040 |
| Sex |  |  |  | 0.342 |
| Female | 324 (38.0%) | 312 (38.3%) | 12 (30.8%) |  |
| Male | 529 (62.0%) | 502 (61.7%) | 27 (69.2%) |  |
| BMI, median (IQR), kg/m^2^ | 22.8 (20.8–25.0) | 22.8 (20.8–25.0) | 22.9 (20.3–25.7) | 0.438 |
| ASA score |  |  |  | 0.025 |
| I | 143 (16.8%) | 137 (16.8%) | 6 (15.4%) |  |
| II | 519 (60.8%) | 502 (61.7%) | 17 (43.6%) |  |
| III | 185 (21.7%) | 169 (20.8%) | 16 (41.0%) |  |
| IV | 6 (0.7%) | 6 (0.7%) | 0 |  |
| Operation method |  |  |  | < 0.001 |
| Open | 571 (66.9%) | 534 (65.6%) | 37 (94.9%) |  |
| Laparoscopy | 181 (21.2%) | 180 (22.1%) | 1 (2.6%) |  |
| Robot | 101 (11.8%) | 100 (12.3%) | 1 (2.6%) |  |
| Surgical procedure |  |  |  | 0.456 |
| STG | 598 (70.1%) | 573 (70.4%) | 25 (64.1%) |  |
| TG | 252 (29.5%) | 238 (29.2%) | 14 (35.9%) |  |
| PG | 3 (0.4%) | 3 (0.4%) | 0 |  |
| Lymph node dissection |  |  |  | 1.000 |
| <D2 | 100 (11.7%) | 96 (11.8%) | 4 (10.3%) |  |
| D2 | 753 (88.3%) | 718 (88.2%) | 35 (89.7%) |  |
| Combined operation |  |  |  | 0.011 |
| No | 731 (85.7%) | 703 (86.4%) | 28 (71.8%) |  |
| Yes | 122 (14.3%) | 111 (13.6%) | 11 (28.2%) |  |
| Histology |  |  |  | 0.444 |
| Differentiated | 266 (31.2%) | 256 (31.4%) | 10 (25.6%) |  |
| Undifferentiated | 587 (68.8%) | 558 (68.8%) | 29 (74.4%) |  |
| Tumor depth |  |  |  | 0.280 |
| T1 | 54 (6.3%) | 52 (6.4%) | 2 (5.1%) |  |
| T2 | 120 (14.1%) | 116 (14.3%) | 4 (10.3%) |  |
| T3 | 308 (36.1%) | 294 (36.1%) | 14 (35.9%) |  |
| T4a | 361 (42.3%) | 344 (42.3%) | 17 (43.6%) |  |
| T4b | 13 (1.2%) | 8 (1.0%) | 2 (5.1%) |  |
| Lymph node metastasis |  |  |  | 0.580 |
| N0 | 207 (24.3%) | 201 (24.7%) | 6 (15.4%) |  |
| N1 | 168 (19.7%) | 159 (19.5%) | 9 (23.1%) |  |
| N2 | 220 (25.8%) | 208 (25.6%) | 12 (30.8%) |  |
| N3 | 258 (30.2%) | 246 (30.2%) | 12 (30.8%) |  |
| Pathologic stage^a^ |  |  |  | 0.254 |
| II | 426 (49.9%) | 410 (50.4%) | 16 (41.0%) |  |
| III | 427 (50.1%) | 404 (42.4%) | 23 (59.0%) |  |

*SC, serious complications; CD, Clavien-Dindo; IQR, interquartile range; BMI, body mass index (calculated as weight in kilograms divided by height in meters squared); ASA, American Society of Anesthesiology; STG, subtotal gastrectomy; TG, total gastrectomy; PG, proximal gastrectomy.*

*^a^Pathologic stages were defined in accordance with the 8^th^ edition of American Joint Committee on Cancer staging system.*

**Table 4.** Comparison of clinicopathologic characteristics between patients with a hospital stay of 15 days or longer with a Clavien-Dindo grade I/II and those with a Clavien-Dindo grade III or higher

|  | **Overall (n=125)** | **Hospital stay ≥15 days with CD I/II (n=39)** | **CD grade III or higher (n=86)** | **P-value** |
| --- | --- | --- | --- | --- |
| Age, median (IQR), year | 66 (54–74) | 64 (56–71) | 67 (54–74) | 0.324 |
| Sex |  |  |  | 0.743 |
| Female | 36 (28.8%) | 12 (30.8%) | 24 (27.9%) |  |
| Male | 89 (71.2%) | 27 (69.2%) | 62 (72.1%) |  |
| BMI, median (IQR), kg/m^2^ | 23.3 (20.6–25.4) | 22.9 (20.3–25.7) | 23.5 (21.0–25.2) | 0.393 |
| ASA score |  |  |  | 0.330 |
| I | 16 (12.8%) | 6 (15.4%) | 10 (11.6%) |  |
| II | 67 (53.6%) | 17 (43.6%) | 50 (58.1%) |  |
| III | 40 (32.0%) | 16 (41.0%) | 24 (27.9%) |  |
| IV | 2 (1.6%) | 0 | 2 (2.3%) |  |
| Operation method |  |  |  | 0.282 |
| Open | 109 (87.2%) | 37 (94.9%) | 72 (83.7%) |  |
| Laparoscopy | 9 (7.2%) | 1 (2.6%) | 8 (9.3%) |  |
| Robot | 7 (5.6%) | 1 (2.6%) | 6 (7.0%) |  |
| Surgical procedure |  |  |  | 0.297 |
| STG | 68 (54.4%) | 25 (64.1%) | 43 (50.0%) |  |
| TG | 56 (44.8%) | 14 (35.9%) | 42 (48.8%) |  |
| PG | 1 (0.8%) | 0 | 1 (1.2%) |  |
| Lymph node dissection |  |  |  | 0.459 |
| <D2 | 9 (7.2%) | 4 (10.3%) | 5 (5.8%) |  |
| D2 | 116 (92.8%) | 35 (89.7%) | 81 (94.2%) |  |
| Combined operation |  |  |  | 0.540 |
| No | 85 (68.0%) | 28 (71.8%) | 57 (66.3%) |  |
| Yes | 40 (32.0%) | 11 (28.2%) | 29 (33.7%) |  |
| Histology |  |  |  | 0.037 |
| Differentiated | 49 (39.2%) | 10 (25.6%) | 39 (45.3%) |  |
| Undifferentiated | 76 (60.8%) | 29 (74.4%) | 47 (54.7%) |  |
| Tumor depth |  |  |  | 0.895 |
| T1 | 4 (3.2%) | 2 (5.1%) | 2 (2.3%) |  |
| T2 | 14 (11.2%) | 4 (10.3%) | 10 (11.6%) |  |
| T3 | 47 (37.6%) | 14 (35.9%) | 33 (38.4%) |  |
| T4a | 55 (44.0%) | 17 (43.6%) | 38 (44.2%) |  |
| T4b | 5 (4.0%) | 2 (5.1%) | 3 (3.5%) |  |
| Lymph node metastasis |  |  |  | 0.794 |
| N0 | 25 (20.0%) | 6 (15.4%) | 19 (22.1%) |  |
| N1 | 28 (22.4%) | 9 (23.1%) | 19 (22.1%) |  |
| N2 | 33 (26.4%) | 12 (30.8%) | 21 (24.4%) |  |
| N3 | 39 (31.2%) | 12 (30.8%) | 27 (31.4%) |  |
| Pathologic stage^a^ |  |  |  | 0.834 |
| II | 53 (42.4%) | 16 (41.0%) | 37 (43.0%) |  |
| III | 72 (57.6%) | 23 (59.0%) | 49 (57.0%) |  |

*CD, Clavien-Dindo; IQR, interquartile range; BMI, body mass index (calculated as weight in kilograms divided by height in meters squared); ASA, American Society of Anesthesiology; STG, subtotal gastrectomy; TG, total gastrectomy; PG, proximal gastrectomy.*

*^a^Pathologic stages were defined in accordance with the 8^th^ edition of American Joint Committee on Cancer staging system.*

**Table 5.** Relationship between postoperative complications and adjuvant chemotherapy in stage II gastric cancer

| **Adjuvant chemotherapy** | **Overall (N=463)** | **Non-SC group (N=410)** | **SC group (N=53)** | **P-value** |
| --- | --- | --- | --- | --- |
| Omission | 463 |  |  | 0.047 |
| (-) | 333 (71.9%) | 301 (73.4%) | 32 (60.4%) |  |
| (+) | 130 (28.1%) | 109 (26.6%) | 21 (39.6%) |  |
| Time to AC initiation | 333 |  |  | 0.027 |
| ≤ 8 weeks | 320 (96.1%) | 292 (97.0%) | 28 (87.5%) |  |
| > 8 weeks (Delay) | 13 (3.9%) | 9 (3.0%) | 4 (12.5%) |  |
| Scheduled cycles of AC | 333 |  |  | 0.078 |
| Completed | 268 (80.5%) | 246 (81.7%) | 22 (68.8%) |  |
| Not completed | 65 (19.5%) | 55 (18.3%) | 10 (31.3%) |  |
| Adequacy of AC | 463 |  |  | 0.004 |
| Adequate^a^ | 261 (56.4%) | 241 (58.8%) | 20 (37.7%) |  |
| Inadequate^b^ | 202 (43.6%) | 169 (41.2%) | 33 (62.3%) |  |

*AC, adjuvant chemotherapy; SC, serious complications.*

*^a^ Adequate AC was defined when AC was completed without omission or delayed initiation.*

*^b^ Inadequate AC was defined when AC was omitted, delayed in initiation, or not completed the scheduled chemotherapy cycles.*

**Table 6.** Relationship between postoperative complications and adjuvant chemotherapy in stage III gastric cancer

| **Adjuvant chemotherapy** | **Overall**  **(N=476)** | **Non-SC group (N=404)** | **SC group (N=72)** | **P-value** |
| --- | --- | --- | --- | --- |
| Omission | 476 |  |  | < 0.001 |
| (-) | 417 (87.6%) | 365 (90.3%) | 52 (72.2%) |  |
| (+) | 59 (12.4%) | 39 (9.7%) | 20 (27.8%) |  |
| Time to AC initiation | 417 |  |  | 0.017 |
| ≤ 8 weeks | 408 (97.8%) | 360 (98.6%) | 48 (92.3%) |  |
| > 8 weeks (Delay) | 9 (2.2%) | 5 (1.4%) | 4 (7.7%) |  |
| Scheduled cycles of AC | 417 |  |  | 0.001 |
| Completed | 315 (75.5%) | 285 (78.1%) | 30 (57.7%) |  |
| Not completed | 102 (24.5%) | 80 (21.9%) | 22 (42.3%) |  |
| Adequacy of AC | 476 |  |  | < 0.001 |
| Adequate^a^ | 309 (64.9%) | 282 (69.8%) | 27 (37.5%) |  |
| Inadequate^b^ | 167 (35.1%) | 122 (30.2%) | 45 (62.5%) |  |

*AC, adjuvant chemotherapy; SC, serious complications.*

*^a^ Adequate AC was defined when AC was completed without omission or delayed initiation.*

*^b^ Inadequate AC was defined when AC was omitted, delayed in initiation, or not completed the scheduled chemotherapy cycles.*

**Table 7.** Comparison of adequacy of adjuvant chemotherapy between the non-SC group and the patients with a hospital stay of 15 days or longer with Clavien-Dindo I/II in stage II/III gastric cancer

| **Adjuvant chemotherapy** | **Overall (n=853)** | **Non-SC group (n=814)** | **Hospital stay ≥15 days with CD I/II (n=39)** | **P-value** |
| --- | --- | --- | --- | --- |
| Omission | 853 |  |  | 0.018 |
| (-) | 692 (81.1%) | 666 (81.8%) | 26 (66.7%) |  |
| (+) | 161 (18.9%) | 148 (18.2%) | 13 (33.3%) |  |
| Time to AC initiation | 692 |  |  | 0.118 |
| ≤ 8 weeks | 676 (97.7%) | 652 (97.9%) | 24 (92.3%) |  |
| > 8 weeks (Delay) | 16 (2.3%) | 14 (2.1%) | 2 (7.7%) |  |
| Scheduled cycles of AC | 692 |  |  | 0.077 |
| Completed | 548 (79.2%) | 531 (79.7%) | 17 (65.4%) |  |
| Not completed | 144 (20.8%) | 135 (20.3%) | 9 (34.6%) |  |
| Adequacy of AC | 853 |  |  | 0.001 |
| Adequate^a^ | 538 (63.1%) | 523 (64.3%) | 15 (38.5%) |  |
| Inadequate^b^ | 315 (36.9%) | 291 (35.7%) | 24 (61.5%) |  |

*AC, adjuvant chemotherapy; SC, serious complications.*

*^a^ Adequate AC was defined when AC was completed without omission or delayed initiation.*

*^b^ Inadequate AC was defined when AC was omitted, delayed in initiation, or not completed the scheduled chemotherapy cycles.*

**Table 8.** Relationship between Clavien-Dindo grade and adjuvant chemotherapy in stage II/III gastric cancer patients with serious complications

| **Adjuvant chemotherapy** | **Overall (n=125)** | **Hospital stay ≥15 days with CD I/II (n=39)** | **CD grade III or higher (n=86)** | **P-value** |
| --- | --- | --- | --- | --- |
| Omission | 125 |  |  | 0.932 |
| (-) | 84 (67.2%) | 26 (66.7%) | 58 (67.4%) |  |
| (+) | 41 (32.8%) | 13 (33.3%) | 28 (32.6%) |  |
| Time to AC initiation | 84 |  |  | 1.000 |
| ≤ 8 weeks | 76 (90.5%) | 24 (92.3%) | 52 (89.7%) |  |
| > 8 weeks (Delay) | 8 (9.5%) | 2 (7.7%) | 6 (10.3%) |  |
| Scheduled cycles of AC | 84 |  |  | 0.660 |
| Completed | 52 (61.9%) | 17 (65.4%) | 35 (60.3%) |  |
| Not completed | 32 (38.1%) | 9 (34.6%) | 23 (39.7%) |  |
| Adequacy of AC | 125 |  |  | 0.893 |
| Adequate^a^ | 47 (37.6%) | 15 (38.5%) | 32 (37.2%) |  |
| Inadequate^b^ | 78 (62.4%) | 24 (61.5%) | 54 (62.8%) |  |

*AC, adjuvant chemotherapy; SC, serious complications.*

*^a^ Adequate AC was defined when AC was completed without omission or delayed initiation.*

*^b^ Inadequate AC was defined when AC was omitted, delayed in initiation, or not completed the scheduled chemotherapy cycles.*

**Figure 1.** Overall (A, C) and recurrence-free survival (B, D) in patients with and without serious complication after gastrectomy by pathologic stage. (A, B) Pathologic stage II. (C, D) Pathologic stage III


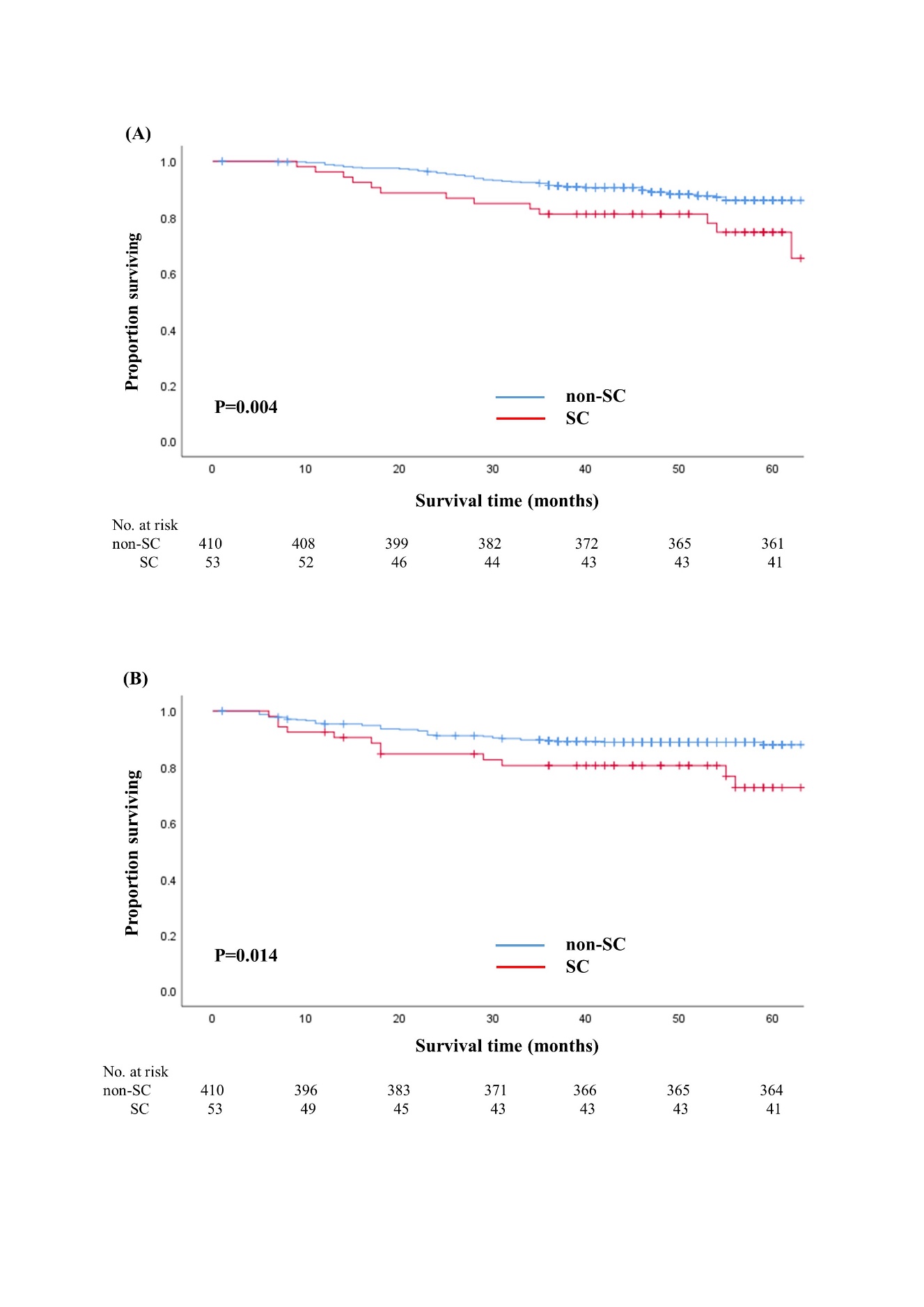


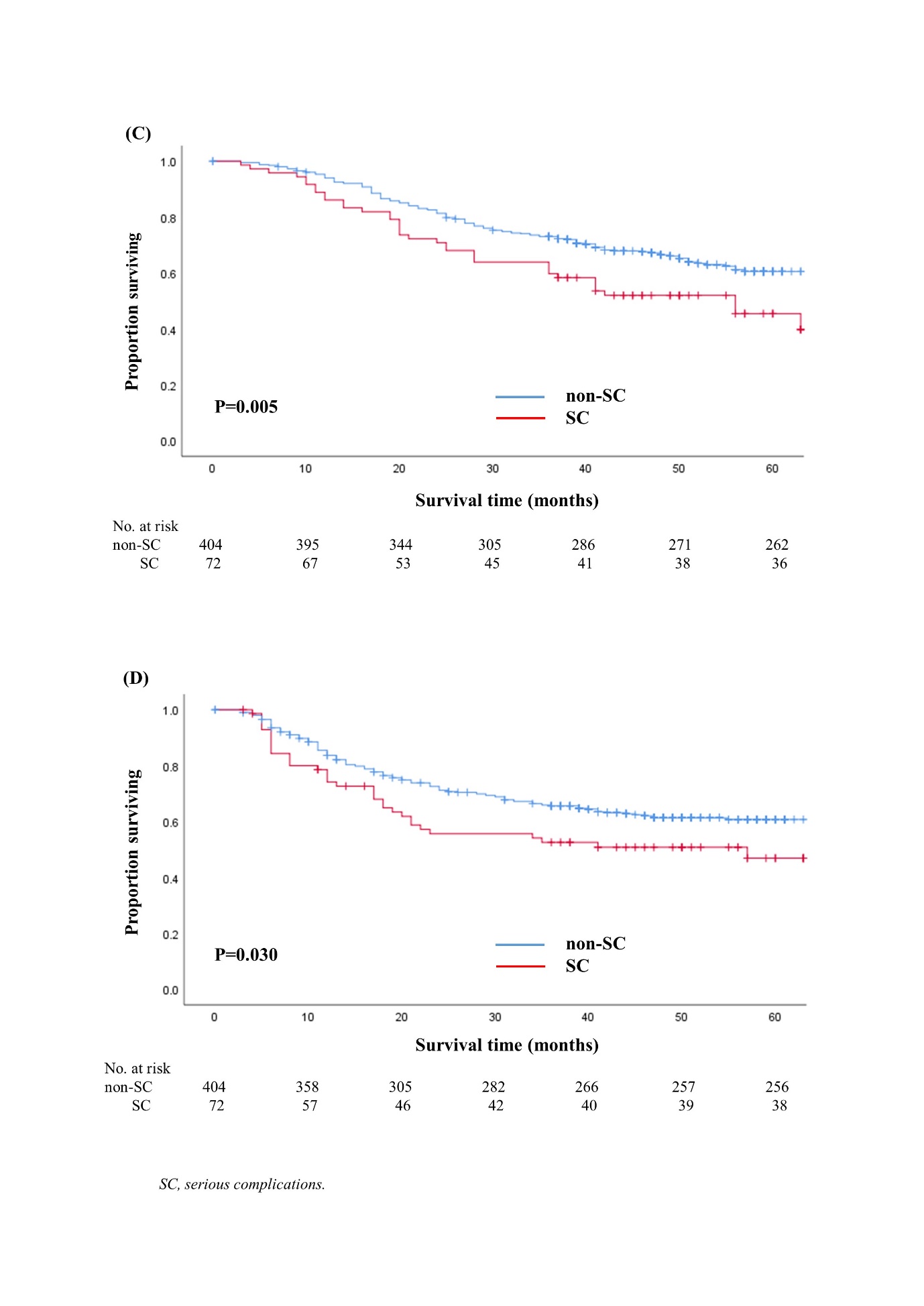


**Figure 2.** Overall (A, C) and recurrence-free survival (B, D) according to the presence of serious complications with the function of adequacy of adjuvant chemotherapy by pathologic stage. (A, B) Pathologic stage II. (C, D) Pathologic stage III


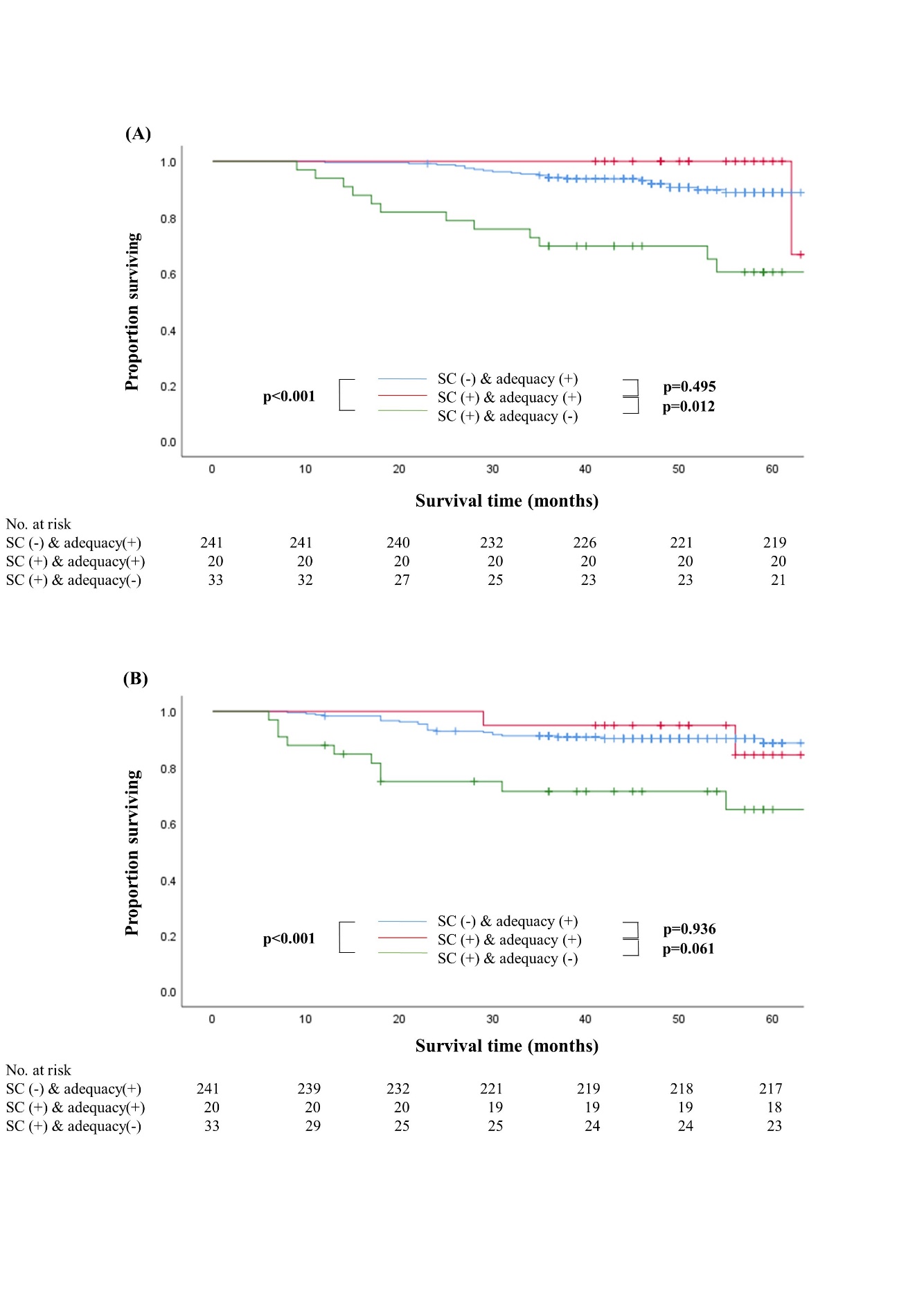


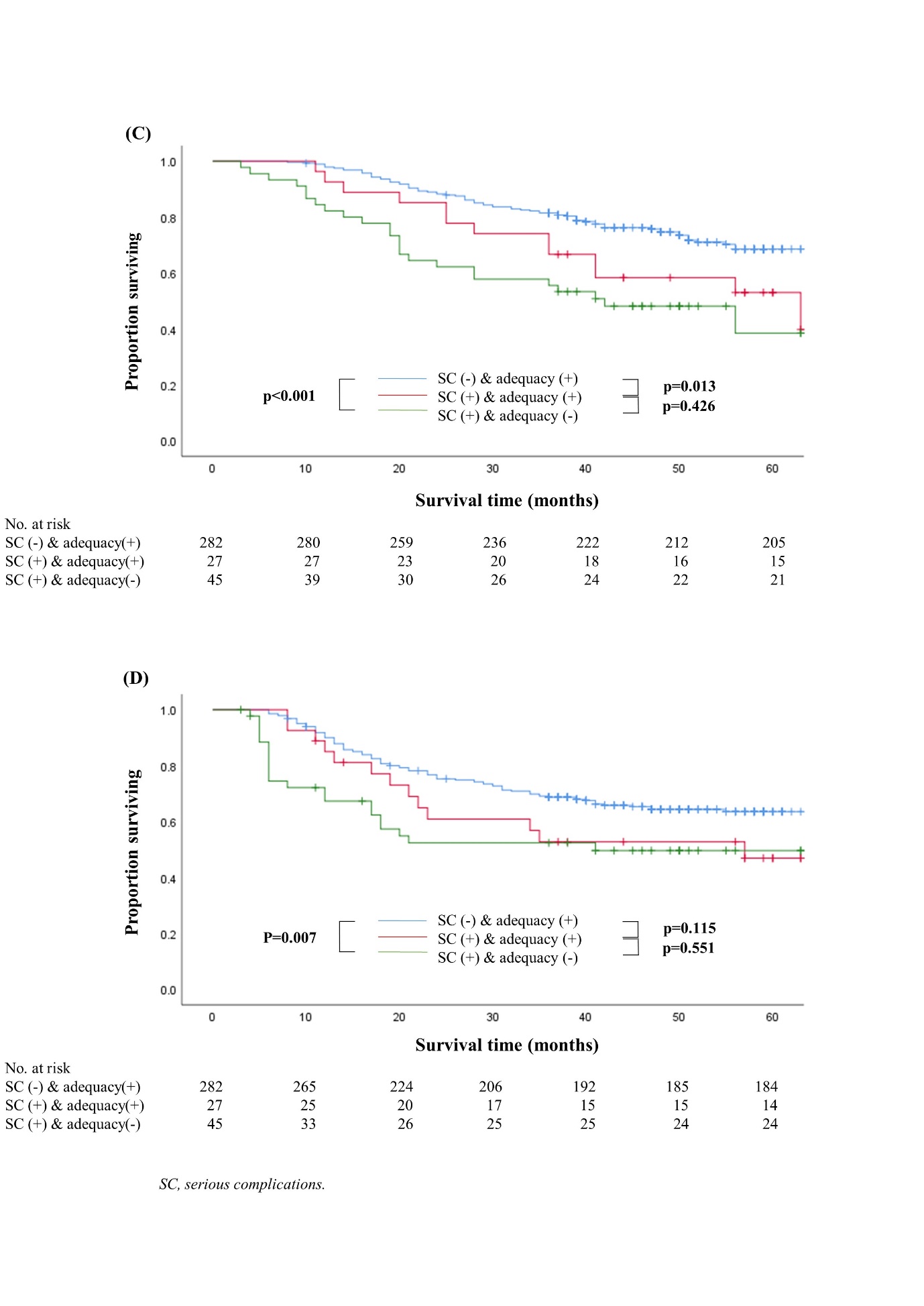


**Figure 3.** Overall (A) and recurrence-free survival (B) in non-SC group and patients with a hospital stay of 15 days or longer with a Clavien-Dindo grade I/II

**
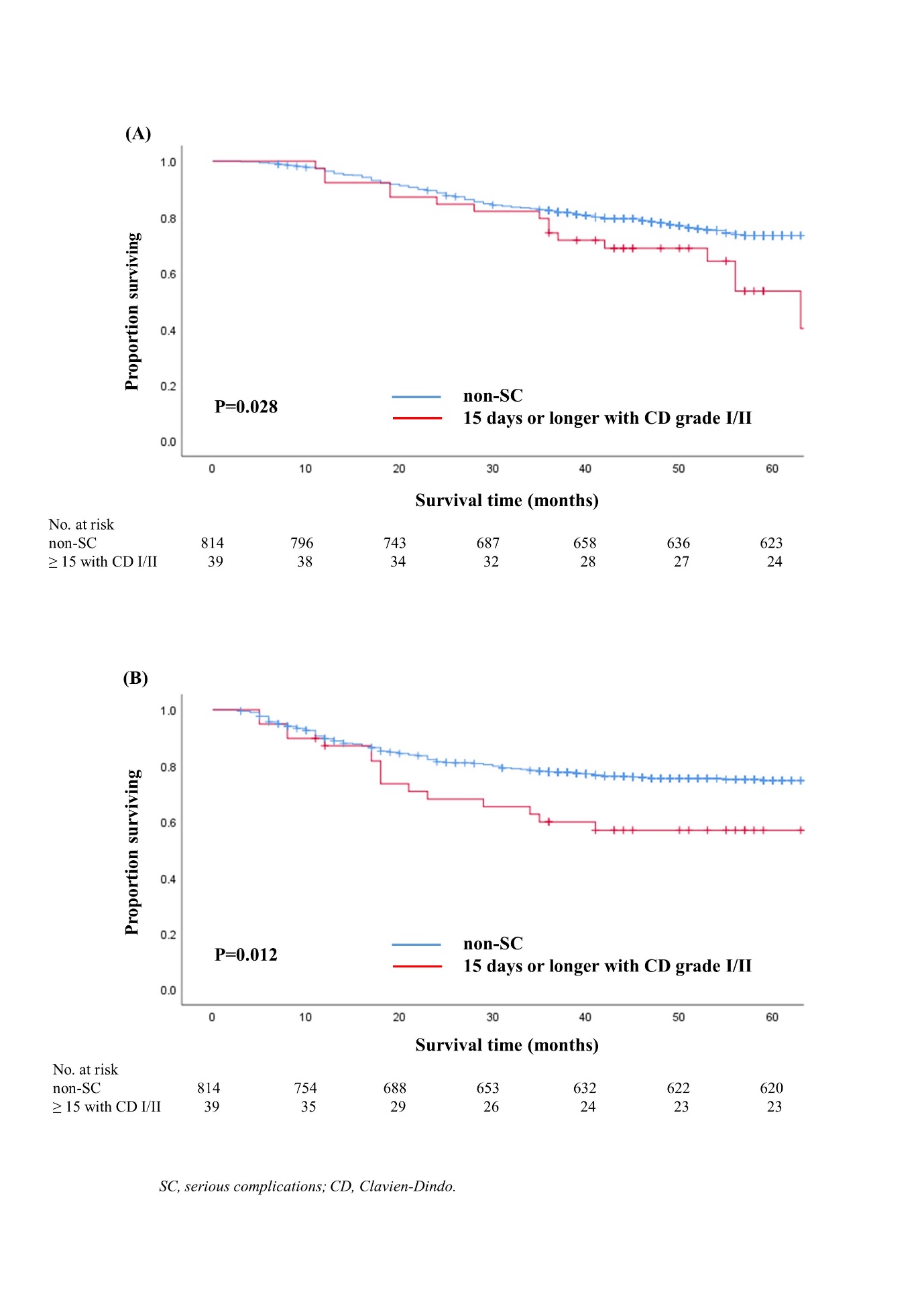
**

**Figure 4.** Overall (A) and recurrence-free survival (B) in patients with Clavien-Dindo grade III or higher and those with a hospital stay of 15 days or longer with a Clavien-Dindo grade I/II


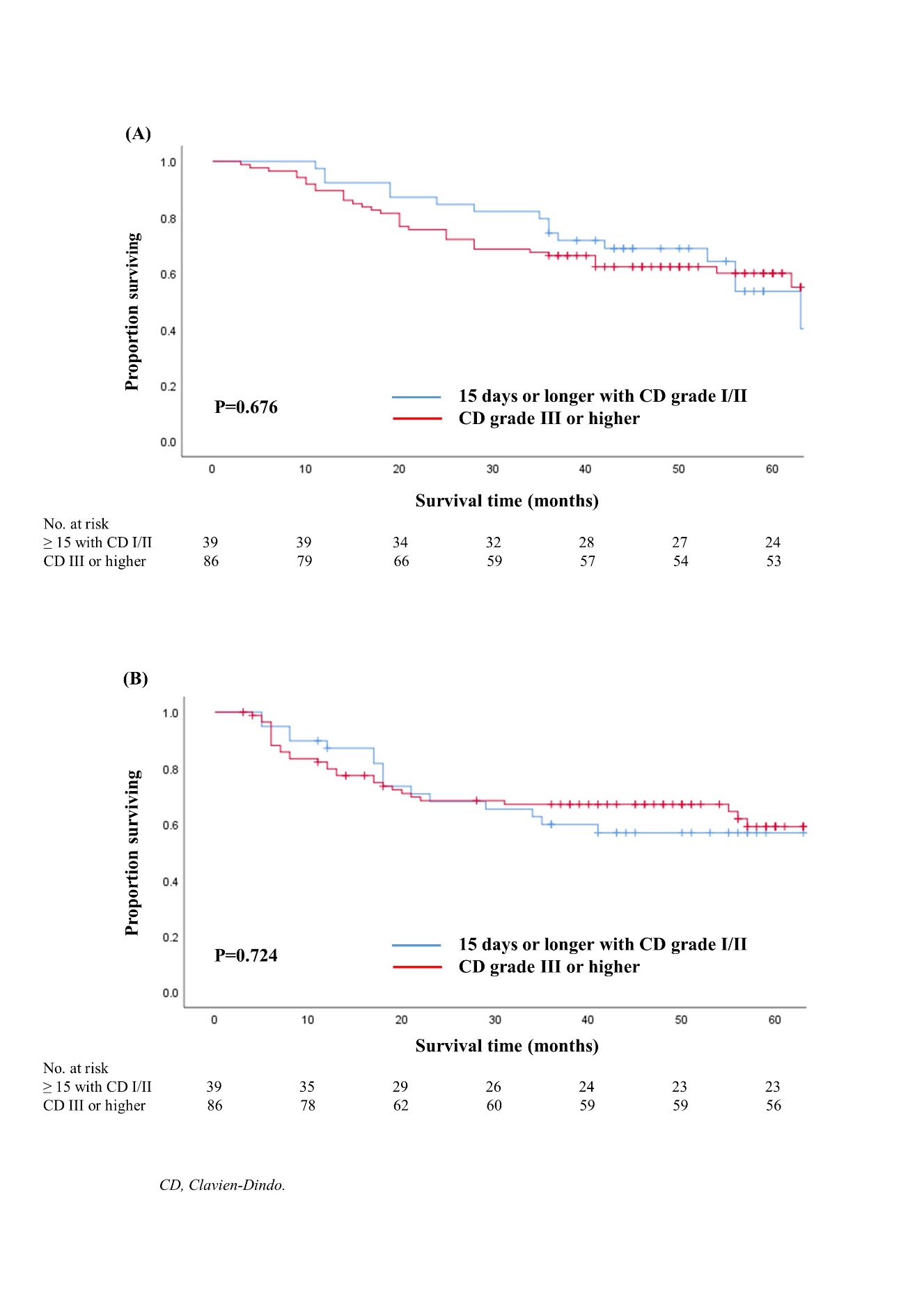


[1] Clavien PA, Barkun J, de Oliveira ML, Vauthey JN, Dindo D, Schulick RD, et al. The Clavien-Dindo classification of surgical complications: five-year experience. Ann Surg 2009;250(2):187-96.
